# Xanthan Gum–Konjac Glucomannan Blend Hydrogel for Wound Healing

**DOI:** 10.3390/polym12010099

**Published:** 2020-01-04

**Authors:** Andreia Alves, Sónia P. Miguel, André R.T.S. Araujo, María José de Jesús Valle, Amparo Sánchez Navarro, Ilídio J. Correia, Maximiano P. Ribeiro, Paula Coutinho

**Affiliations:** 1 CPIRN-IPG- Center of Potential and Innovation of Natural Resources, Polytechnic Institute of Guarda, Av. Dr. Francisco de Sá Carneiro, No. 50, 6300-559 Guarda, Portugal; 2 Department of Pharmaceutical Sciences, Faculty of Pharmacy, University of Salamanca, 37007 Salamanca, Spain; 3 CICS-UBI- Health Sciences Research Centre, University of Beira Interior, Av. Infante D. Henrique, 6200-506 Covilhã, Portugal; 4 LAQV, REQUIMTE, Department of Chemical Sciences, Laboratory of Applied Chemistry, Faculty of Pharmacy, Porto University, Rua de Jorge Viterbo Ferreira, 228, 4050-313 Porto, Portugal; 5 Institute of Biopharmaceutical Sciences of University of Salamanca (IBSAL), 37007 Salamanca, Spain; 6 CIEPQPF, Department of Chemical Engineering, University of Coimbra, P-3030 790 Coimbra, Portugal

**Keywords:** wound dressing, thermo-reversible hydrogel, xanthan gum, konjac glucomannan

## Abstract

Hydrogels are considered to be the most ideal materials for the production of wound dressings since they display a three-dimensional structure that mimics the native extracellular matrix of skin as well as a high-water content, which confers a moist environment at the wound site. Until now, different polymers have been used, alone or blended, for the production of hydrogels aimed for this biomedical application. From the best of our knowledge, the application of a xanthan gum–konjac glucomannan blend has not been used for the production of wound dressings. Herein, a thermo-reversible hydrogel composed of xanthan gum–konjac glucomannan (at different concentrations (1% and 2% *w*/*v*) and ratios (50/50 and 60/40)) was produced and characterized. The obtained data emphasize the excellent physicochemical and biological properties of the produced hydrogels, which are suitable for their future application as wound dressings.

## 1. Introduction

The skin is the largest organ of the human body, with a weight ranging from 3.5–10 kg and occupying an area of 1.5–2.0 m^2^. It plays an important role in organism homeostasis [1]. When skin integrity is compromised, as a consequence of traumatic or chronic events, the wound healing process begins to re-establish the structure and functions of the tissue as soon as possible [2]. In order to improve patients’ odds of survival, and to minimize the loss of skin vital functions, the wound must be covered immediately after an injury occurs [3]. To accomplish this, different dressings have been developed, including hydrocolloids, transparent films, foams, hydrogels, sponges, bandages, etc. These dressings have been developed for the treatment of wounds as well as to provide a protective physical barrier for the wound site from the external environment. However, despite these efforts, none of the wound dressings that have been produced so far are fully capable of mimicking all the natural features of skin tissue.

Among the wound dressings developed, hydrogels have attracted the attention of researchers due to their intrinsic capacity to reproduce the three-dimensional (3D) structure of the skin extracellular matrix. Furthermore, hydrogels are hydrophilic 3D networks that are capable of absorbing significant amounts of water or biological fluids, like wound exudate [4,5]. In addition, hydrogels are able to clean dry, sloughy, or necrotic wounds by rehydrating dead tissues (moist healing), leading to an increment of autolytic debridement and cooling of the surface of the wound. Thus, hydrogels contribute to pain reduction and, consequently, increase patient acceptance of the dressing. Moreover, the biodegradability of hydrogels avoids the complications associated with wound dressing replacement, such as the risk of infection, tissue maceration and pain [6,7].

To accomplish the production of hydrogels, researchers have been using different natural polymers, like alginate [8], chitosan [9], collagen [10], dextran [11], hyaluronan [12], xanthan [13], and konjac [14], among others. Catanzano et al. produced a hydrogel composed of alginate and hyaluronan aiming to improve the wound healing process [8]. These authors verified that the produced hydrogels were able to promote cell migration as well as reduce the area of wounds previously induced on animal models. On the other hand, Miguel et al. developed a thermo-responsive hydrogel composed of chitosan and agarose that displayed excellent biocompatibility and was able to enhance the wound healing process [9].

Herein, a xanthan gum–konjac glucomannan blend was used for the first time to produce a wound dressing that was subsequently evaluated in physicochemical and biological assays. Konjac glucomannan (KGM) is a natural polysaccharide [15] extracted from the tubers of the *Amorphophallus konjac* plant [16]. It is composed of β-1,4-linked d-glucose units with glucose/mannose (G/M), with a G/M ratio around 1:1.6 [17,18,19,20]. KGM has been widely used in the food industry as a gelling agent or thickener due to its biocompatibility and biodegradability [21]. Nevertheless, KGM application in the production of wound dressings is restricted by the weak mechanical properties displayed in aqueous solutions. Such behavior occurs as a consequence of the high number of hydroxyl groups available in its polymeric chain [22]. To overcome this limitation, xanthan gum (XG) was used to upgrade the mechanical strength of the produced hydrogel. XG is a negatively charged and high molecular weight extracellular hetero-polysaccharide, with a cellulose-like backbone, obtained from *Xanthomonas campestris* [23,24,25]. It is composed of 1,4 linked β-d-glucose residues, with a trisaccharide side chain of β-d-mannose β-d-glucuronic acid-α-d-mannose attached to alternate d-glucose units of the main chain [23,24,25]. In general, XG can present approximately 2.54–4.41% acetyl and 6.43% pyruvate groups, which can vary according to the culture conditions [26]. XG, in contact with aqueous solutions, presents two different conformations: an ordered and rigid double helical strand structure at low temperature and a disordered and flexible coil structure at high temperatures [27]. When the temperature of a XG solution is below its midpoint transition temperature (Tm)—about 40–50 °C—the ordered double helical strand structure forms a three-dimensional network, exhibiting a gel-like behavior [21]. This thermal gelation property of XG was already tested by Dyondi et al., who prepared an injectable hydrogel by combining gellan gum with XG which displayed gelation temperature values around 37–40 °C [28]. The produced hydrogel was then applied directly at the desired site without eliciting any harmful side effects [29,30]. Taking into account the features of XG and KGM, these materials were used herein to produce a new in situ forming hydrogel composed of XG and KGM to be applied as a wound dressing.

## 2. Materials and Methods

### 2.1. Materials

Xanthan gum was purchased from Acofarma (*M*_W_ > 1,000,000 Da) (Terrassa, Spain); konjac glucomannan was obtained from Sports Supplemented Ltd. (Colchester, UK); 3-(4,5-dimethyl-2-thiazolyl)-2,5-diphenyl-2H-tetrazolium bromide (MTT) was acquired from VWR (Radnor, PA, USA); dimethyl sulfoxide (DMSO) and L(+)-ascorbic acid were bought from Appli Chem. Panreac (Barcelona, Spain); sodium hydroxide (NaHCO_3_) was purchased from José Manuel Gomes dos Santos, Lda. (Odivelas, Portugal); bovine serum albumin (BSA) was obtained from Biowest; Dulbecco’s modified Eagle’s medium (DMEM)-F12, fetal bovine serum (FBS), phosphate-buffered saline (PBS) and trypsin were purchased from Sigma (Sintra, Portugal); normal human dermal fibroblast (NHDF) cells were bought from PromoCell (Labclinics, S.A., Barcelona, Spain).

### 2.2. Synthesis of XG/KGM Hydrogels

The XG/KGM hydrogels were produced by mixing XG and KGM powder in distilled water at a final concentration of 1% and 2% (*w*/*v*) using different ratios (1%_XG/KGM_(50/50), 1%_XG/KGM_(60/40), 2%_XG/KGM_(50/50), 2%_XG/KGM_(60/40)). The polymeric solutions were homogenized by autoclaving at 121 °C, for 30 minutes. After that, the XG/KGM hydrogels were formed by decreasing the temperature to 37 °C. The produced hydrogels were stored at 3 °C until use.

### 2.3. Structural Analysis and Characterization of XG/KGM Hydrogels

#### 2.3.1. Fourier Transform Infrared Spectroscopy (FTIR) Analysis

FTIR analysis was performed to characterize the composition of the produced hydrogels. The spectra were acquired by using an average of 128 scans, with a spectral width ranging from 400 cm^−1^ to 4000 cm^−1^ and a spectral resolution of 32 cm^−1^. All the samples were mounted on a diamond window and the spectra were recorded using a Nicolet iS10 FTIR spectrophotometer (Thermo Scientific, Waltham, MA, USA). In order to compare the spectra of individual components with those of the produced hydrogels, all the raw components used for hydrogel production were also analyzed [31].

#### 2.3.2. Characterization of the Mechanical Properties

The mechanical properties were determined by using a Texture Analyzer (TA-XT Plus, Stable Micro Systems, Surrey, UK). The assays were performed by using a 35 mm diameter disc that was compressed into the hydrogel and redrawn. The method settings selected were a 5 kg load cell with a penetration depth of 10 mm and a test speed of 2 mm/s. After penetrating the sample, the probe returned to a position 30 mm above the platform surface. From the force (g)/time (s) plot obtained, the parameter firmness (maximum force), cohesiveness (positive area) and adhesiveness (negative area) were determined. The firmness represents the hardness of the hydrogel formulation, whereas the cohesiveness is defined as the work required to deform the hydrogel with the downward movement of the probe [32]. The measurements were performed in quadruplicate at controlled room temperature (RT).

#### 2.3.3. Determination of Moisture Content

Standard International Union of Pure and Applied Chemistry (IUPAC) method was used to evaluate the moisture content. A sample of the hydrogels (W_i_) was taken in a porcelain crucible and heated in a temperature-controlled water bath for about six hours at 105 °C. The sample was then cooled in desiccators and weighed again (W_t_) [13]. The moisture percentage in hydrogels was calculated using the following formula:

Moisture (%) = ((W_i_ − W_t_)/W_i_) × 100 (1)

#### 2.3.4. Study of Water Uptake Capacity (Swelling)

To evaluate the water uptake capacity of XG/KGM hydrogels, a known weight (W_i_) of each hydrogel was immersed in 1 mL Tris buffer at pH 5 and 37 °C (*n* = 5). At predetermined intervals, the swollen samples were removed from the solution, quickly wiped to remove the excess water on the surface and then weighed (W_t_), as previously described in the literature [9].

The water uptake ratio was evaluated using the equation:

Water uptake ratio (%) = ((W_t_ − W_i_)/W_i_) × 100 (2)

#### 2.3.5. Determination of the Contact Angle

The water contact angle of hydrogels was determined using a data physics contact angle system OCAH 200 apparatus (DataPhysics Instruments GmbH, Filderstadt, Germany) operating in static mode at room temperature. For each sample, water drops were placed at various locations on the hydrogel surface [33]. The determined contact angles are the average of at least three measurements.

#### 2.3.6. Surface Morphology Characterization

XG/KGM hydrogel morphologies were characterized by scanning electron microscopy (SEM). The samples were frozen (−80 °C) and then freeze-dried at −110 °C and lower pressure for 3 h. After that, samples were mounted onto aluminum stubs and sputter-coated with gold using a Quorum Q150R ES sputter coater (Quorum Technologies Ltd., Laughton, East Sussex, UK). The SEM images were acquired at variable magnifications using an acceleration voltage of 20 kV with a Hitachi S-3400N scanning electron microscope (Hitachi, Tokyo, Japan) [31]. 

### 2.4. Characterization of the Biological Performance of the Produced Hydrogels

#### 2.4.1. Proliferation and Adhesion of Fibroblasts in the Presence of XG/KGM Hydrogels

To evaluate the growth of human fibroblast cells in the presence of XG/KGM hydrogel, they were seeded in 96-well plates with DMEM-F12, containing the sterilized hydrogels (20 µL/well) using a cellular density of 3 × 10^4^ cells/well, and incubated at 37 °C in a 5% CO_2_ humidified atmosphere for 24 h and 72 h. Ethanol (96%) was added to cells to be used as positive controls (K^+^), whereas cells without biomaterials were used as negative controls (K^−^). Cell growth was monitored with an Optika inverted light microscope equipped with an Optikam B5 digital camera (Bergamo, Italy).

Additionally, using the same cell density (3 × 10^4^ cells/well), fibroblast cells adhesion to the hydrogel surface was also characterized by SEM. The samples were fixed with glutaraldehyde (2.5% (*v*/*v*)) overnight and then analyzed by SEM as previously described in Section 2.3.6.

#### 2.4.2. Characterization of the Cytotoxic Profile of XG/KGM Hydrogels

The cytotoxic profile of the hydrogels was characterized using an 3-(4,5-dimethylthiazol-2-yl)-2,5-diphenyltetrazolium bromide (MTT) assay that was performed according to the guidelines set by the International Organization for Standardization (ISO) 10993-5 standard. Briefly, 20 µL of XG/KGM hydrogel was placed in each well of a sterile 96 well plate, and then human fibroblast cells were seeded at a density of 3 × 10^4^ cells/well and incubated at 37 °C in a 5% CO_2_ humidified atmosphere for 24 h and 72 h. The medium was then removed and 50 µL of MTT (5 mg/mL PBS) was added to each sample (*n* = 5), followed by incubation for 4 h at 37 °C in a 5% CO_2_ atmosphere. Then, cells were treated with 200 µL of DMSO (0.04 N) for 30 min. A microplate reader (Biorad xMark microplate spectrophotometer, Waltham, MA, USA) was used to read the absorbance at 570 nm of the samples from each well. Cells cultured without materials were used as a negative control (K^−^), whereas cells cultured with EtOH (96%) were used as a positive control (K^+^).

#### 2.4.3. Fibroblast Distribution within XG/KGM Hydrogels

Confocal laser scanning microscopy (CLSM) was used to visualize the cell distribution within hydrogels. Cells (1.6 × 10^4^ cells/well) were seeded in hydrogels in μ-Slide 8-well Ibidi imaging plates (Ibidi GmbH, Planegg/Martinsried, Germany) on the surface of the hydrogel. After 24 h and 72 h, the nucleus of the cells were stained with Hoechst 33342 (2 μM, Thermo Fisher Scientific, Waltham, MA, USA), whereas the hydrogels were labelled with calcein (40 μg/mL). Then, the imaging experiments were performed using a Zeiss LSM710 laser scanning confocal microscope (Carl Zeiss AG, Oberkochen, Germany), where consecutive z-stacks were acquired. The 3D reconstruction and image analysis were performed using Zeiss Zen 2010 software [33].

#### 2.4.4. Migration of Fibroblasts in a Wound Healing Scratch Assay

Cell migration was evaluated through a scratch wound healing assay as described by Jonkman et al. [34], where 2.5 × 10^5^ cells/well were seeded in a 24-well plate with 1 mL of DMEM-F12 until confluence was attained. Then, a linear scratch wound was generated in the monolayer with a sterile 20 µL plastic tip. Any cellular debris was removed by washing the plate with PBS. Then, 100 µL of hydrogel and 1mL of fresh medium were added to the cultures, which were then incubated at 37 °C inside an incubator with a 5% CO_2_ humidified atmosphere for 24 h. Cell migration was determined after 0 h, 3 h, 6 h, 9 h, 12 h and 24 h using an Optika inverted light microscope equipped with an Optikam B5 digital camera (Bergamo, Italy). The area of cell migration into the wound site was quantified using ImageJ (Scion Corp., Frederick, MD, USA) and presented as a relative migration compared with t = 0, which was considered as 100% [34].

### 2.5. Statistical Analysis

The statistical analysis of the obtained results was performed using one-way analysis of variance (ANOVA) with Tukey’s post hoc test. A *p*-value lower than 0.05 (*p* < 0.05) was considered statistically significant.

## 3. Results

### 3.1. Characterization of the Morphologic and Physicochemical Properties of XG/KGM Hydrogels

**Figure 1:**
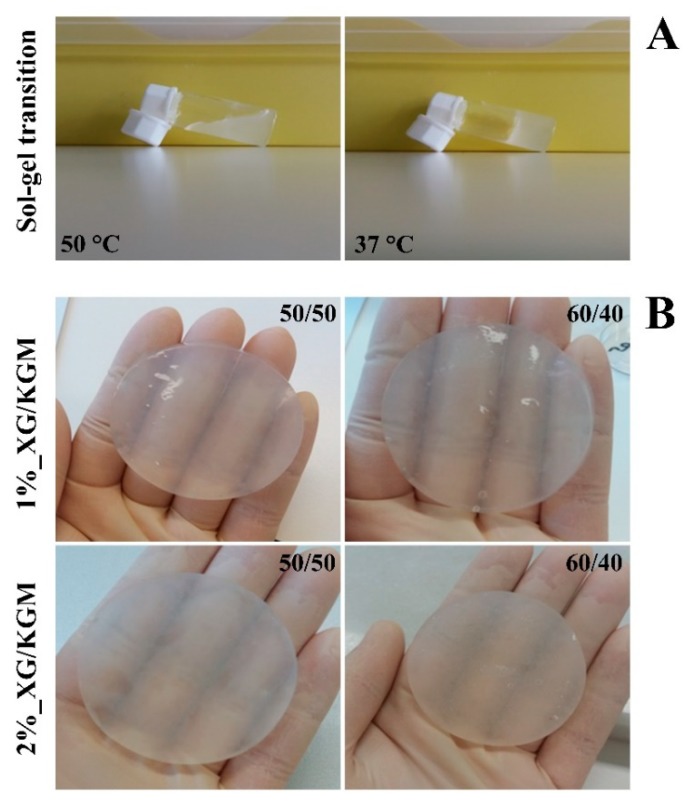
The solid and the gel states of hydrogels observed at different temperatures (**A**) and the macroscopic images of the hydrogels produced in this study (**B**) XG/KGM: xanthan gum–konjac glucomannan.

Hydrogels are known for exhibiting a 3D network quite similar to that found in native skin, as well as by presenting a high content of water. These properties are essential for these dressings being capable of enhancing the healing process [13,35]. In this study, the XG/KGM hydrogels were obtained when the solution temperature value was decreased to 37 °C or below, as represented in Figure 1A. The thermo-responsive character of hydrogels results from the presence of XG, which has a gel-like behavior at temperatures around 37 °C [27]. This property allowed the production of an in situ forming hydrogel that can be directly applied at the wound site and that can acquire the shape and size of the wound. 

Furthermore, it is possible to see in Figure 1B that the produced hydrogels display a transparent and moisturized appearance, which allows the continuous monitoring of the wound healing process without requiring dressing removal. On the other hand, the high moisture content of hydrogels enables a cooling effect at the wound, which reduces the pain and improves patient acceptability [36,37].

**Figure 2:**
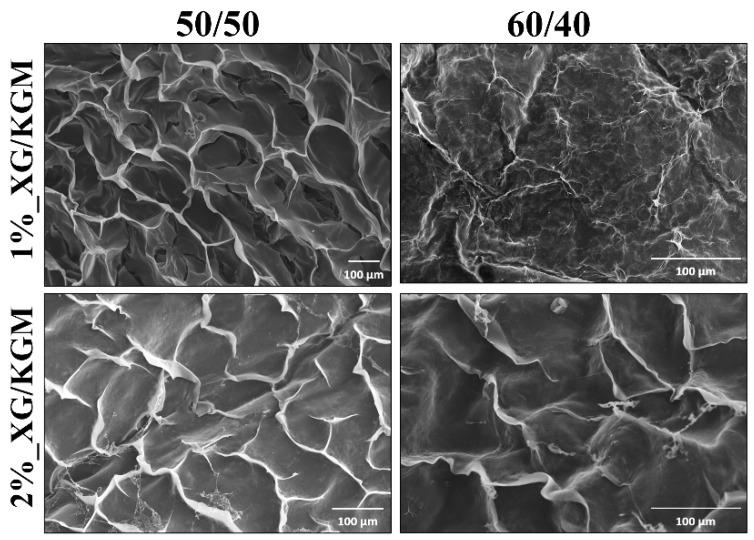
Representative scanning electron microscopy (SEM) images of the different ratios and percentages of XG/KGM used to produce hydrogels. Scale bar: 100 μm.

Apart from the easy conformability of hydrogels at the wound site, the morphological features of its surface play a crucial role in the interaction with surrounding tissues. In this way, the surface morphology of XG/KMG hydrogels was characterized by SEM analysis. The obtained SEM images (Figure 2) showed that all formulations presented an irregular and rough surface, which is favorable for cell recruitment/adhesion, proliferation, differentiation, and ultimately skin regeneration improvement [22]. Indeed, the rough surfaces exhibit a higher surface area and topographical motifs that constitute anchoring points for cells or proteins that mediate fibroblast adhesion [11]. Deligianni et al. have already demonstrated that the cell adhesion, proliferation and differentiation is enhanced at rough surfaces [38].

**Figure 3:**
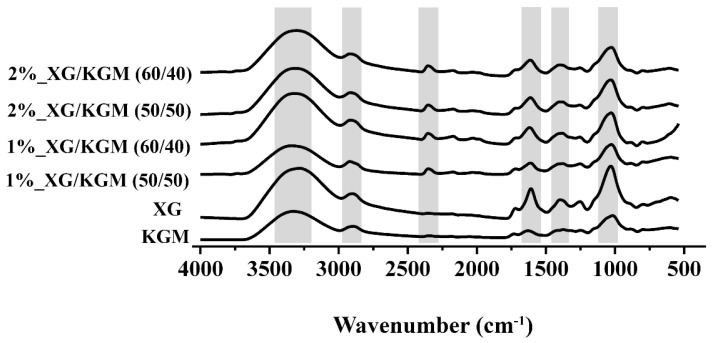
FTIR spectra of the individual components (XG and KGM) and of the different blends (1%_XG/KGM_(50/50), 1%_XG/KGM_(60/40), 2%_XG/KGM_(50/50) and 2%_XG/KGM_(60/40)) used for the production of hydrogels.

Moreover, the chemical composition of the hydrogels was characterized through FTIR analysis in order to identify the conformational changes and intermolecular interaction of each polymer in the hydrogel formulation (see Figure 3 for further details). The KGM and XG spectra exhibited peaks at 3300 cm^−1^ (–OH), 2910 cm^−1^ (C–H bond of the macromolecule hexatomic ring), 1620 cm^−1^ (band of carbonyl of acetyl groups), 1240 cm^−1^ (C–O stretching) and at 1410 cm^−1^ (–CH_2_). Furthermore, these peaks and an additional peak at 2349 cm^−1^ (O=C=O stretching) were also observed in the hydrogel spectra.

Additionally, in the hydrogel spectra, the characteristic peaks of both KGM and XG presented a shift to lower wavenumbers, which was more noticeable for the band located at 1630–1620 cm^−1^. Such an effect may be explained by the establishment of new hydrogen bonds between XG and KGM [16,39,40].

### 3.2. Determination of Firmness, Cohesiveness and Adhesiveness of XG/KGM Hydrogels

An ideal wound dressing must have optimal mechanical properties (hardness and cohesiveness) and bio-adhesion (prolonged contact time at a wound site) in order to act as a stable protective barrier and simultaneously interact with the cell components present at the wound site [41]. 

The results obtained in these assays are summarized in Table 1, demonstrating that higher polymer concentrations lead to an increase of the firmness and cohesiveness of hydrogel formulations. Furthermore, the change in polymer ratio from 50/50 to 60/40 resulted in a decrease of firmness and cohesiveness values. Such results evidenced that the polymer concentration and the amount of gelling agent (XG) have a direct impact on the mechanical properties of hydrogels. This influence was already evidenced by Hurler et al. [32], who verified that an increase in polymer concentration and gelling agent prompted an increase of the hardness and cohesiveness of Carbopol and poloxamer hydrogels. Similarly, Karavana et al. found an increase in gel cohesiveness with an increase in hydroxypropylmethylcellulose concentration [42] and Cevher et al. reported identical observations for Carbopol gels [43].

In relation to the adhesiveness values obtained in this study (Table 1), it is possible to notice that all produced hydrogels exhibited similar results, confirming their excellent bio-adhesion. In turn, the adhesiveness reflects the ability of hydrogel formulations to interact with surrounding tissues [44,45]. These data are in agreement with those previously reported in the literature, where bio-adhesive ability was associated with the presence of hydrophilic polymers [32,46,47]. In fact, the presence of hydroxyl, amino and carboxyl groups at a material’s surface promotes protein adsorption, providing optimal conditions for cell adhesion and proliferation [48]. Considering the obtained results, all hydrogel formulations presented the firmness, cohesiveness and adhesiveness required to be applied as a wound dressing.

### 3.3. Characterization of Water Content, Water Uptake Profile and Wettability of XG/KGM Hydrogels

Enhanced cell proliferation in moist environments was demonstrated for the first time by George Winter when he discovered that the epithelialization process occurred twice fast in moist environments in comparison to the dry environments [49]. In this way, it is crucial to develop wound dressings able to provide suitable moisture for cell adhesion and proliferation [50]. 

**Figure 4:**
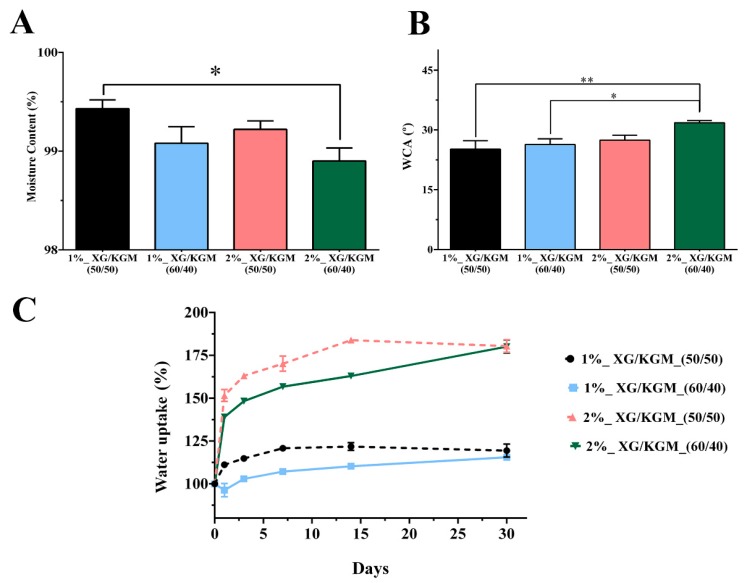
Physicochemical characterization of the hydrogels’ properties. (**A**) Moisture content. (**B**) Water contact angle (WCA). (**C**) Water uptake assay. *, ** Significant differences between groups. The data are shown as means ± standard deviations (*n* = 3).

In this study, the water content of hydrogels was determined (Figure 4A) and the results revealed that all the different blends of XG/KGM had a water content higher than 99%. Such results confirm the suitability of XG/KGM hydrogels to provide moist environments as well as prevent the dehydration of the wound [51,52].

Additionally, the hydrophilicity of the XG/KGM hydrogel surface was also evaluated by determining the water contact angles. As shown in Figure 4B, all XG/KGM formulations had water contact angle values below 90°, confirming their hydrophilic character, which is considered beneficial for cell adhesion [31].

However, it is worth noticing that an excess of humidity at the wound site may lead to the formation of excessive exudate. The abnormal accumulation of wound exudate can cause tissue maceration, impair cell migration and hinder the healing process. Therefore, it is crucial that wound dressings provide a moist environment and are simultaneously able to absorb excess wound exudate.

Therefore, the water uptake profile of hydrogels was evaluated and the results (Figure 4C) revealed that 1% XG/KMG (1%_XG/KGM (50/50) and 1%_XG/KGM (60/40)) exhibited a lower water uptake ability in comparison to the 2% XG/KMG hydrogels (2%_XG/KGM (50/50) and 2%_XG/KGM (60/40)). Such differences were observed since a higher content of polysaccharides (XG/KGM) leads to the presence of more hydrophilic compounds (hydroxyl and carboxyl groups), which can easily interact with water molecules and consequently increase the water uptake [9,44,45]. Previous studies presented similar results for other polysaccharides-based hydrogels [53,54]. Overall, all the produced hydrogels were able to provide a moist environment and improve cell adhesion (due to their hydrophilic character) while avoiding the accumulation of exudate at the wound site.

### 3.4. Characterization of the Biological Properties of XG/KMG Hydrogels

#### 3.4.1. Evaluation of the Cytotoxic Profile of the Hydrogels

The biocompatibility of the produced hydrogels was evaluated through their incubation with fibroblasts cells. These cells were used as a model since they play an important role in the production of extracellular matrix (ECM) components (collagen and fibronectin), cytokines and growth factors that are essential for tissue repair [31].

**Figure 5:**
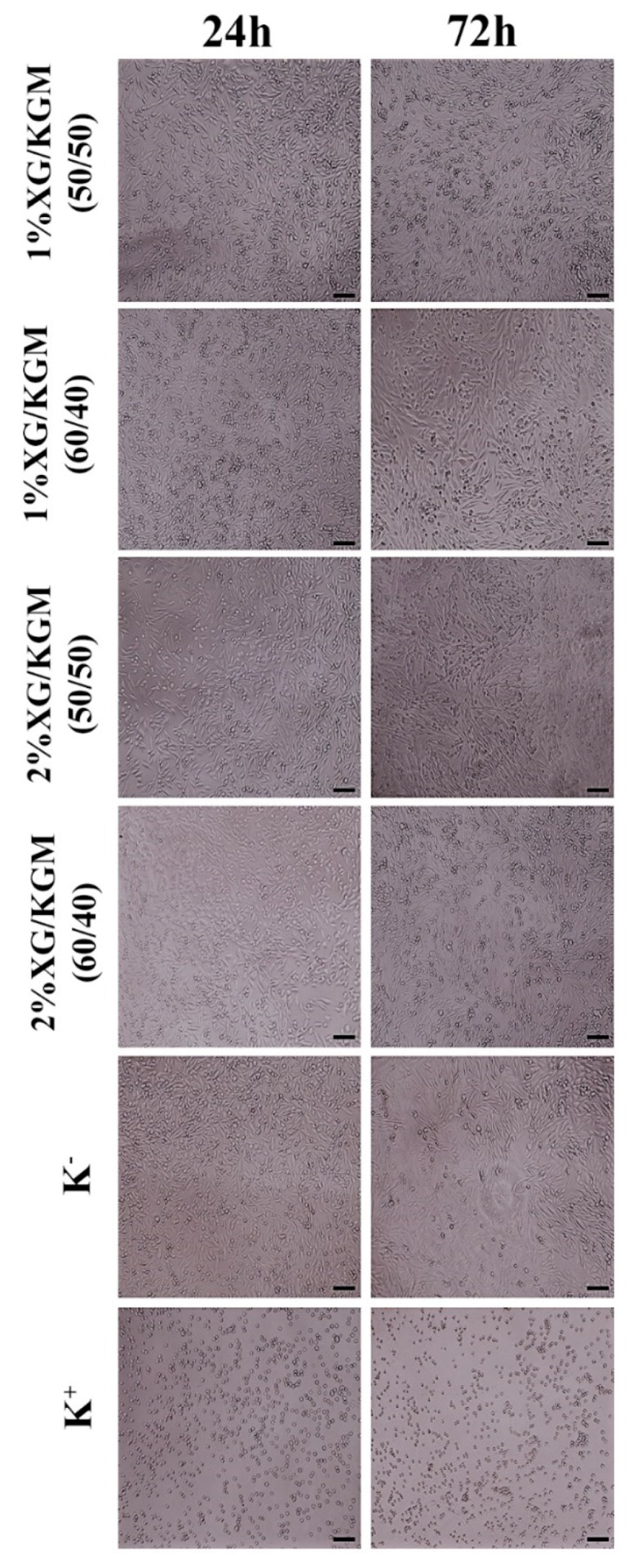
Microscopic images of human fibroblast cells in contact with XG/KMG hydrogels after 24 h and 72 h. K^−^ (negative control), K^+^ (positive control). Scale bar: 100 µm.

The microscope images (Figure 5) showed that the morphology and proliferation of fibroblasts were not affected by the presence of hydrogels for 24 h and 72 h. These cells displayed similar features to those available on the negative control (cells seeded in contact with culture medium).

**Figure 6:**
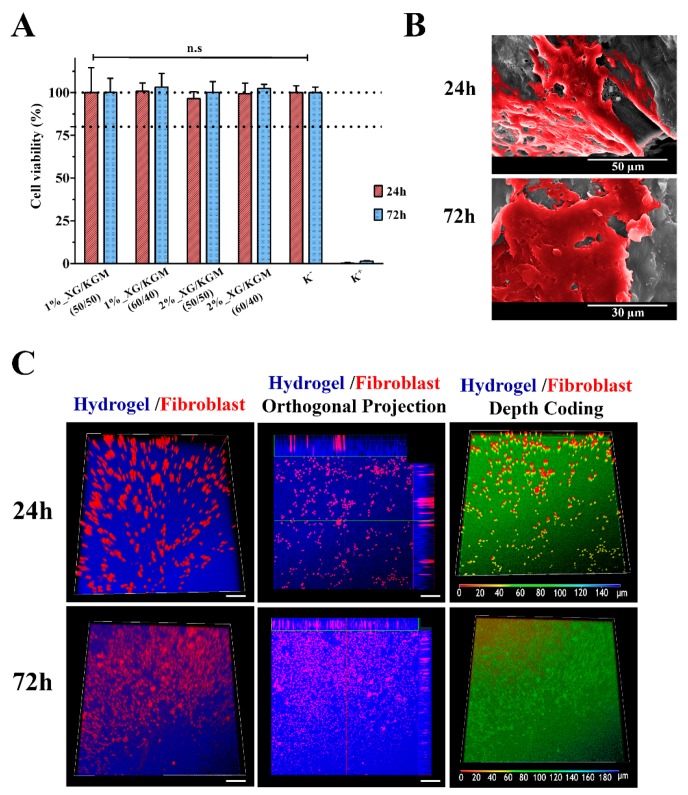
Characterization of the cytocompatibility of the hydrogels. (**A**) MTT assay of human fibroblast cells grown in the presence of different hydrogels. Wells treated with ethanol were used as positive controls. n.s: no statistically significant groups. The data are shown as means ± standard deviations (*n* = 3). (**B**) Representative SEM images of fibroblast cell adhesion and proliferation on the surface of the 1%_XG/KGM_(60/40) hydrogel, after 24 h and 72 h of incubation. (**C**) Confocal laser scanning microscopy (CLSM) images of cell internalization in 1%_XG/KGM_(60/40) after 24 h and 72 h, where different colors correspond to distinct depth values (as indicated in the color-coding scale).

Further, the cytotoxic profile of the XG/KGM hydrogels was also assessed by MTT assay after 24 h and 72 h. The metabolic conversion of MTT to purple formazan crystals occurs in living cells and is proportional to the number of viable cells present in each well. The results obtained in the MTT assay (Figure 6A) showed that fibroblast cell viability was not compromised during the 24 h and 72 h that cells were in contact with XG/KGM hydrogels. Such results were expected since the biocompatibility of XG and KGM-based hydrogels was previously reported in the literature [55,56,57]. 

#### 3.4.2. Characterization of Cell Adhesion and Infiltration into Hydrogels

Apart from having a biocompatible profile, a wound dressing must also act as a 3D scaffold that is able to support cellular attachment, growth and migration. Hence, cell interaction with the hydrogel surface was evaluated through SEM analysis. Figure 6B showed that the hydrophilic character and roughness of the hydrogel surface was suitable for cell adhesion and proliferation. In fact, after 72 h, it can be observed that the cells presented with typical fibroblastic morphology; interconnections between cells were established that led to the formation of a continuous cell layer [58].

In addition, fibroblast migration and proliferation within XG/KGM hydrogels was also characterized by confocal laser scanning microscopy (CLSM) analysis. The CLSM images (Figure 6C) showed that cells were able to migrate into the inner structure of XG/KGM hydrogels. Cellular internalization was also noticeable in the orthogonal projection. Further, the depth color coding images also confirmed that the fibroblast cells migrated within the hydrogels during the incubation period. The majority of cells remained at a depth of 0–20 µm after 24 h (which appear in red), whereas after 72 h, the cells appeared at a depth of 80–120 µm (which appear in green).

#### 3.4.3. In Vitro Wound Healing Assay

In order to study the effect of XG/KGM hydrogels on fibroblast migration, a scratch wound healing assay was performed following a procedure reported by Gabbiani et al. [59]. The migration of fibroblast cells was measured using an image analysis software after 3 h, 6 h, 9 h, 12 h and 24 h and compared to 0 h.

**Figure 7:**
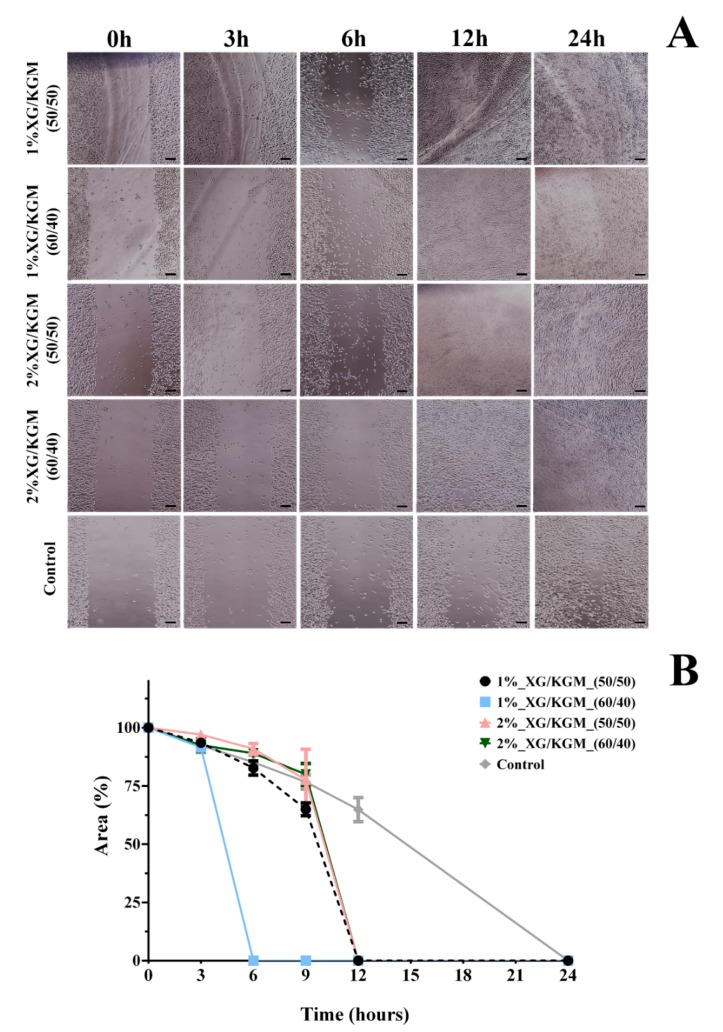
Cell migration response to biomaterial conditioned medium. (**A**) Fibroblast migration in the presence of hydrogels formulations and only culture medium (control). (**B**) Effect of the different hydrogels on the migratory activities of fibroblasts in the scratch assay. Data are expressed as a percentage of cell area compared to the control. Scale bar: 100 µm.

Through the analysis of the results presented in Figure 7, it is possible to see that the 1%_XG/KGM_(50/50), 2%_XG/KGM_(50/50) and 2%_XG/KGM_(60/40) hydrogels promoted full cover of the wound site after 12 h, whereas the wound area was filled only after 24 h for the control group. Even more, 1%_XG/KGM_(60/40) promoted an improved and quick wound closure in comparison to all formulations, which occurred after 6 h.

Such results highlight that the products resulting from XG/KGM polysaccharide degradation (e.g., residues of glucose, mannose, glucuronic acid and glucomannan) encourage cell migration. Indeed, according to Shahbuddin et al., the glucomannan interacts with growth factor receptors available on fibroblast membranes, stimulating their activity and proliferation [55,60]. Further, Al-Ghazzewi et al. also reported that the mannose-rich carbohydrates (such XG) enhance the wound healing process [61].

All together, these results demonstrate the potential of XG/KGM hydrogels to improve the wound healing process by promoting fibroblast migration, adhesion and proliferation. When the fibroblasts are present at the wound site, they produce ECM components (namely collagen type III) and secrete growth factors (such as fibroblast and vascular endothelial growth factors) involved in the reestablishment of the structure of the damaged skin [62,63].

## 4. Conclusions

Hydrogels are known as ideal wound dressings since they possess intrinsic properties that favor cell adhesion, migration and proliferation, thus encouraging the healing process. More recently, in situ wound dressings have received special attention from researchers due to their ability to be directly applied at the wound site and their ability to acquire the required size and shape of the wound. In this work, an in situ thermo-reversible hydrogel was produced through the combination of two polysaccharides (XG and KGM) without the use of chemical crosslinkers. To the best of our knowledge, this combination has not been used previously to accomplish the production of a wound dressing. All data gathered herein demonstrated that XG/KGM hydrogels are hydrophilic and able to provide a moist environment while absorbing excess exudate. In addition, the hydrogels also possessed adequate biological properties for supporting cell adhesion, migration and proliferation. 

In the near future, other complementary in vitro assays (e.g., evaluation of the viscoelastic properties, porosity, and biodegradability) may be performed in order to fully depict the potential of hydrogels for the treatment of skin injuries. Additionally, the incorporation of antimicrobial molecules (e.g., silver nanoparticles and/or natural extract-derived products) can be hypothesized to further improve the wound healing process, as well as avoiding the occurrence of skin infections.

## Figures and Tables

**Table 1:** Texture analysis of XG/KGM hydrogels.

Hydrogel	Firmness (g)	Cohesiveness (g.s)	Adhesiveness (g.s)
1%_ XG/KGM_(50/50)	3816 ± 160	16,474 ± 1075	−4253 ± 558
1%_ XG/KGM_(60/40)	2364 ± 170	11,536 ± 334	−2879 ± 176
2%_ XG/KGM_(50/50)	4156 ± 325	19,762 ± 1425	−2554 ± 527
2%_ XG/KGM_(60/40)	3265 ± 159	15,456 ± 1129	−2950 ± 703
